# Benefit Cost Analysis of Three Skin Cancer Public Education Mass-Media Campaigns Implemented in New South Wales, Australia

**DOI:** 10.1371/journal.pone.0147665

**Published:** 2016-01-29

**Authors:** Christopher M. Doran, Rod Ling, Joshua Byrnes, Melanie Crane, Anthony P. Shakeshaft, Andrew Searles, Donna Perez

**Affiliations:** 1 School of Human Health and Social Sciences, Central Queensland University, Brisbane, Queensland, Australia; 2 Hunter Medical Research Institute, University of Newcastle, Newcastle, New South Wales, Australia; 3 Centre for Applied Health Economics, Griffith University, Brisbane, Queensland, Australia; 4 Cancer Institute New South Wales, Sydney, New South Wales, Australia; 5 School of Public Health, University of Sydney, Sydney, New South Wales, Australia; 6 National Drug and Alcohol Research Centre, University of New South Wales, Sydney, Australia; A*STAR, SINGAPORE

## Abstract

Public education mass media campaigns are an important intervention for influencing behaviour modifications. However, evidence on the effectiveness of such campaigns to encourage the population to reduce sun exposure is limited. This study investigates the benefits and costs of three skin cancer campaigns implemented in New South Wales from 2006–2013. This analysis uses Australian dollars (AUD) and 2010–11 as the currency and base year, respectively. Historical data on skin cancer were used to project skin cancer rates for the period 2006–2020. The expected number of skin cancer cases is derived by combining skin cancer rates, sunburn rates and relative risk of skin cancers due to sun exposure. Counterfactual estimates are based on sunburn exposure in the absence of the campaigns. Monetary values are attached to direct (treatment) and indirect (productivity) costs saved due to fewer skin cancer cases. Monetary benefits are compared with the cost of implementing the campaigns and are presented in the form of a benefit-cost ratio. Relative to the counterfactual (i.e., no campaigns) there are an estimated 13,174 fewer skin cancers and 112 averted deaths over the period 2006–2013. The net present value of these benefits is $60.17 million and the campaign cost is $15.63 million. The benefit cost ratio is 3.85, suggesting that for every $1 invested a return of $3.85 is achieved. Skin cancer public education mass media campaigns are a good investment given the likely extent to which they reduce the morbidity, mortality and economic burden of skin cancer.

## Introduction

Skin cancer is one of the most common cancers in the world [1–6]. In Australia, melanoma, basal cell carcinoma (BCC) and squamous cell carcinoma (SCC) are the skin cancer types diagnosed most frequently [3]. Australia has the world’s highest age-standardised rate of melanoma (37 per 100,000 persons), more than 12 times the average world rate in 2008 (3 per 100,000 persons) [3]. BCC and SCC are commonly referred to as non-melanoma skin cancer (NMSC). Both melanoma and NMSC are highly preventable and are caused by overexposure to harmful ultraviolet radiation (UVR) [7]. The most effective means of reducing risk of developing skin cancer is to avoid direct exposure to UVR during the time of day when solar UVR levels are moderate to extreme and to avoid using solaria [3].

Preventive strategies such as sunscreen or mass media campaigns have only recently been evaluated using techniques of economic evaluation [8–10]. Gordon et al (2009) estimated the cost-effectiveness of a skin cancer prevention initiative based on regular sunscreen use. Compared with usual practice (discretionary use), the sunscreen intervention cost an additional US$106,449 to prevent 35 NMSCs and 838 actinic keratoses among 812 residents over 5 years. These health outcomes required an annual average investment of US$0.74 per person and saved the Australian government a total of US$88,203 in healthcare costs over the same period [8]. Hirst et al (2012) investigated the lifetime health costs and benefits of sunscreen promotion in the primary prevention of skin cancer. The authors reported that over the projected lifetime of the intervention cohort, the intervention would prevent 33 melanomas, 168 SCCs and 4 melanoma-deaths at a cost of approximately AUD$808,000 [9]. Shih et al (2009) [10] examined the cost-effectiveness of the SunSmart program, which has primarily used school-based education and mass media campaign activities [11]. The authors estimated 9,000 melanomas, 94,000 NMSCs and over 1,000 deaths were prevented in Victoria from 1988 to 2003 due to the program [10].

Prior to the implementation of the Skin Cancer Prevention Strategy in 2012, three specific campaigns by the Cancer Institute of New South Wales (CINSW) formed the basis of skin cancer public education mass media activities in NSW. The Tattoo campaign was implemented first in 2006–2007 (following its implementation in several other Australian other states) as a joint initiative of CINSW and Cancer Council NSW. In the summer of 2007–2008 the Dark Side of Tanning (DSOT) campaign was launched with the first of three commercials focused on addressing perceptions about tanning and the connection between tanning and development of melanoma. These commercials specifically targeted adolescents and young adults with one theme and message line, ‘there’s nothing healthy about a tan’. The Wes Bonny Testimonial campaign followed in 2011–2013, supporting the repeat of the DSOT campaign with a testimonial story of Wes Bonny, a young man who died from melanoma. The campaign included testimonials by the parents, brothers and friends of Wes Bonny and focused on the risk of melanoma. Television was the primary medium for these campaigns. Given these three campaigns are similar, in that they all targeted UVR exposure, tanning and sun protection attitudes, this study estimates the potential benefits and costs (expressed as a benefit-cost ratio (BCR)) of their combined effect.

## Method

### Ethics

Ethics clearance was obtained from the New South Wales Ministry of Health (2012/09/417). Patient or any other type of informed consent was not required for this study as the project relied on historical data from linked administrative data sets. All patient level information was de-identified.

### Key data sources

#### Target audience rating points (TARPs)

TARPs are the broadcasting metrics used to measure viewing exposure calculated as the average viewing audience exposed to the advertisement (reach) as a proportion of the target population (frequency) for a television (TV) commercial campaign. These measures are based on Australian TV audience measurements for adults aged 18 years and older for free-to-air and cable TV. Commencing in summer 2006/07, weekly summer TARPs data were recorded for the three campaigns included in this analysis. Although TARPs are measured each week, the effect of TV campaigns may have an accumulation, lagged or decay effect. For example, a decay effect occurs when previous exposure to TV commercials impacts on current behaviour but with a diminished effect-size relative to the period immediately after initial exposure. A TARPs rating for Sydney metropolitan with a decay of 4 weeks was chosen as the preferred measure of exposure in this analysis given the fact that the National Sun Protection Survey sample for NSW was drawn mainly from metropolitan residents and this measure has been used in other studies [12, 13].

#### National Sun Protection Survey (NSPS)

The NSPS is a cross-sectional survey of Australian residents aged 12–69 years coordinated by the Anti-Cancer Council of Victoria and funded by all Australian Cancer Councils. The survey is conducted by telephone over 8 weeks of summer and includes questions about tanning, sunburn and sun protection attitudes, beliefs, behaviour and social norms. NSPS NSW data were collected from 4,966 participants in total over 3 waves: 2003–04, 2006–07 and 2010–11. A booster sample of <25 year olds was collected for wave 3 in 2010–11 by CINSW. For all three survey waves, there were 8 interview weeks over each summer, giving a total of 24 interview weeks.

#### Melanoma: historical and projected rates

Historical data on cases of melanoma were sourced from the NSW Central Cancer Registry (CCR) for the period 1995 to 2008 (Table A in S1 File) [14]. To take account of changes in skin cancer incidence by age, four age cohorts were considered—less than 25 years (<25), 25–49 years, 50–74 years and older than 75 years (75+). Cohort specific rates (crude cohort rates) were calculated using CINSW guidelines [15] and NSW population estimates [16]. Historical trends were used to develop regression equations which were then used to project cohort-specific rates for the period 2006–2020. The modelled projections were validated by actual observed data for the period 2006 to 2008.

#### Non melanoma skin cancer (NMSC)

Unlike other invasive cancers, NMSC is not notifiable by law to cancer registries in Australia [3]. Data on rates of NMSC from the 2002 National Cancer Control Initiative survey [17] were combined with NSW population estimates [16] to estimate the cohort specific incidence and cases of NMSC in 2006. Projections for the period 2006 to 2020 were made using historical melanoma trends.

### Effectiveness of the campaign on behaviour change

Annual sunburn rates were estimated for all cohorts using data from the NSPS and TARP. Expected sunburn rates (i.e., sunburn rates in the presence of the campaigns) were derived using the average campaign exposure (measured by TARPs). Counterfactual sunburn rates (i.e., sunburn rates in the absence of the campaigns) were derived by assuming no campaign exposure (measured by setting TARPs equal to zero). Based on these data, regression equations were used to project sunburn rates (expected and counterfactual) for each cohort from 2006 to 2020.

### Impact of the campaign on skin cancer incidence and mortality

Compared to people who report never having been sunburnt, self-reported lifetime sunburn is associated with an increased relative risk (RR) of self-reported melanoma (RR = 1.91; 95% CI = 1.69–2.17), BCC (RR = 1.71; 95% CI = 1.54–1.90) and SCC (RR = 1.23; 95% CI = 0.90–1.69) [18]. Consequently, the expected number of skin cancer cases in any year can be calculated by combining cohort specific rates, expected sunburn rates in the population and the relative risk of skin cancers among those who report having been sunburned and those who do not. The counterfactual estimate is derived using the same method but by replacing expected sun burn rates with counterfactual sun burn rates. The difference in expected and counterfactual provide an estimate of the overall effectiveness of the campaign in averting new cases of skin cancer.

The effects of the campaigns are assumed to coincide with duration, i.e., 2006–2013. We test in the sensitivity analysis the assumption that the campaign will have residual effects until 2020, at which time the expected sunburn rates would converge with the counterfactual sunburn rates. The inclusion of a time lag between sunburning and a diagnosis of skin cancer would also be feasible. Given there is no consensus on the appropriate length of this lag, the benefits of the public education mass media campaigns are assumed to be immediate, which is consistent with Shih et al’s (2009) economic evaluation of the SunSmart program [10]. This assumption is also tested in a sensitivity analysis by adopting a 5 year lag on campaign effectiveness. Analysis of lags of 10 or 15 years would require extended modelling of skin cancer incidence past 2020, creating additional uncertainty.

Data from the Australian Institute of Health and Welfare suggest that the case fatality rate for melanoma and NMSC is 12.3% (1,544 deaths / 12,510 cases) and 0.13% (543 deaths / 433,613 cases), respectively. To calculate an estimate of deaths averted, these mortality rates are multiplied with the number of averted melanoma and SCC cases.

### Economic analysis

#### Economic value of skin cancer incidence and mortality

Doran et al (2015) estimated the economic costs of skin cancer in NSW for 2010 [19]. Briefly, an incidence based approach was used to estimate lifetime costs of skin cancer. Both direct and indirect costs are considered—direct costs include resources associated with the management of skin cancer and indirect costs refer to productivity costs associated with morbidity and premature mortality. Diagnosis of skin cancer was determined according to ICD-10 codes using principal diagnosis. Linked administrative data and regression modelling are used to calculate costs; presented as Australian dollars for the year 2010. Estimation of lifetime direct costs used longitudinal methods to estimate average costs for each diagnosis group, for each calendar year since diagnosis—for 5 years post diagnosis for melanoma and 2 years post diagnosis for NMSC. A cost per annum from year of diagnosis reflects that any cost difference between those with and without a diagnosis is likely to be most pronounced in the first years of diagnosis. Regression analysis is used to estimate the effect on the cost associated with a diagnosis of either melanoma or NMSC, compared to the control group, i.e., those without a diagnosis. The human capital approach is used to value present and future productivity losses. Table B in S1 File provides a summary of the key economic parameters used in the current analysis.

#### Benefit-cost analysis (BCA)

This analysis uses 2010–11 as the reference year and adopted a societal perspective in conducting a BCA. In a BCA, a ratio can be created by dividing benefits over costs, referred to as a benefit-cost ratio (BCR). A ratio greater than 1 demonstrates a positive return on investment. In this analysis the potential benefits are measured as treatment and productivity costs avoided due to reducing the incidence of, and mortality from, skin cancer. The costs include the CINSW campaign cost. All future costs are converted to present value using a 3% discount rate (i.e., to reflect the opportunity cost of money based on the official cash rate) [20].

### Sensitivity analysis

The sensitivity of the BRC to variations in key assumptions are explored by: applying the lower confidence limits of the RR between sunburn and skin cancer; by extending the potential benefits of the campaign to the period 2006–2020; and, by lagging potential benefits of the campaign by a 5 year period (i.e., 2011–2020).

## Results

### Impact of the campaigns on behaviour change

Table 1 provides information on sunburn rates (expected and counterfactual) for each cohort over the period 2006–2020. Sun burn rates are generally higher for the younger cohorts and are projected to decline across all cohorts. The rate of decline is greater due to exposure to the campaigns, and, in the absence of additional campaign funding, is assumed to converge by 2020.

**Table 1:** Expected and counterfactual sunburn rates by cohort, 2006–2020.

Year	<25 expected	<25 CF	25–49 expected	25–49 CF	50–74 expected	50–74 CF	75+ expected	75+ CF
2006	21.7%	22.9%	14.7%	20.6%	10.5%	12.7%	6.5%	10.7%
2007	20.7%	22.0%	13.6%	20.1%	9.7%	12.1%	6.0%	9.4%
2008	19.9%	21.3%	12.8%	19.6%	9.1%	11.6%	5.7%	8.7%
2009	19.2%	20.7%	12.1%	19.3%	8.6%	11.1%	5.4%	8.2%
2010	18.7%	20.2%	11.6%	19.0%	8.2%	10.8%	5.1%	7.9%
2011	18.2%	19.8%	11.1%	18.8%	7.8%	10.5%	4.9%	7.6%
2012	17.8%	19.5%	10.7%	18.5%	7.5%	10.3%	4.7%	7.4%
2013	17.4%	19.1%	10.4%	18.3%	7.3%	10.0%	4.6%	7.2%
2014	17.4%	18.8%	11.4%	18.2%	7.5%	9.8%	4.8%	7.0%
2015	17.5%	18.6%	12.4%	18.0%	7.8%	9.6%	5.1%	6.9%
2016	17.5%	18.3%	13.3%	17.8%	8.0%	9.5%	5.4%	6.8%
2017	17.5%	18.1%	14.3%	17.7%	8.2%	9.3%	5.6%	6.7%
2018	17.5%	17.9%	15.3%	17.6%	8.5%	9.2%	5.9%	6.6%
2019	17.5%	17.7%	16.3%	17.4%	8.7%	9.1%	6.1%	6.5%
2020	17.5%	17.5%	17.3%	17.3%	8.9%	8.9%	6.4%	6.4%

Note: CF = counterfactual. CF is without campaign and expected is with campaign.

### Impact of the campaigns on averting skin cancer incidence and mortality

Relative to the counterfactual (i.e., no campaigns) there are an estimated 885 fewer cases of melanoma and 12,289 fewer cases of NMSC (10,159 BCC and 2,130 SCC) over the period 2006–2013 (Table 2 and Tables C–E in S1 File). For melanoma and NMSC, this is equivalent to 111 and 1,536 fewer cases each year, respectively. An estimated 109 (12.3%*885) melanoma and 2.7 (0.13%*2,130) SCC deaths are averted as a consequence of the campaigns.

**Table 2:** Skin cancer cases averted, by age group, 2006–2013.

	<25				25–49					50–74				75+				All Ages			
Year	Mel	BCC	SCC	Total	Mel	BCC	SCC	Total	Total	Mel	BCC	SCC	Total	Mel	BCC	SCC	Total	Mel	BCC	SCC	Total
2006	0	1	0	1	35	365	58	458	34	34	353	97	483	14	227	43	284	83	946	198	1,226
2007	0	1	0	1	38	406	64	509	38	38	396	109	542	16	259	49	324	93	1,062	222	1,376
2008	0	1	0	2	42	440	70	551	41	41	433	119	593	18	286	54	357	101	1,160	243	1,503
2009	1	1	0	2	44	469	74	588	45	45	466	128	639	19	310	59	388	109	1,247	261	1,617
2010	1	1	0	2	47	493	78	618	48	48	498	137	683	21	334	63	419	116	1,327	278	1,721
2011	0	1	0	2	49	513	81	642	51	51	529	145	725	22	359	68	449	122	1,402	294	1,818
2012	0	1	0	2	50	532	84	666	53	53	558	153	764	24	382	72	479	128	1,473	310	1,910
2013	0	1	0	2	52	550	87	689	56	56	586	161	803	25	406	77	508	134	1,543	325	2,002
***Total***	***4***	***9***	***0***	***12***	***357***	***3*,*769***	***595***	***4*,*721***	***365***	***365***	***3*,*819***	***1*,*049***	***5*,*232***	***160***	***2*,*563***	***485***	***3*,*208***	***885***	***10*,*159***	***2*,*130***	***13*,*174***

Note: totals may not add due to rounding;

Mel = melanoma;

BCC = basal cell carcinoma;

SCC = squamous cell carcinoma.

### Economic value of averting skin cancer incidence and mortality

The total combined economic value of averting 13,174 skin cancers and 112 deaths is estimated at $67.55 million over the period 2006–13 (equivalent to a net present value of $60.17 million) (Table 3 and Tables F-H in S1 File). Treatment savings accounted for 56% ($37.76 million) and productivity savings 44% ($29.79 million) of the total economic value.

**Table 3:** Economic value of averting skin cancer cases and deaths, 2006–2013.

Year	Direct cost savings	Indirect cost savings—morbidity	Indirect cost savings—premature mortality	Total indirect cost savings	Total
2006	$3,518,531	$2,264,307	$523,218	$2,787,525	$6,306,056
2007	$3,947,754	$2,537,503	$586,311	$3,123,814	$7,071,568
2008	$4,311,821	$2,769,102	$639,798	$3,408,899	$7,720,720
2009	$4,635,167	$2,974,255	$687,171	$3,661,426	$8,296,593
2010	$4,932,987	$3,161,550	$730,403	$3,891,954	$8,824,940
2011	$5,210,888	$3,334,938	$770,411	$4,105,349	$9,316,237
2012	$5,472,549	$3,498,107	$808,059	$4,306,166	$9,778,715
2013	$5,732,549	$3,660,070	$845,426	$4,505,496	$10,238,045
***Total***	***$37*,*762*,*244***	**$24,199,831**	**$5,590,799**	***$29*,*790*,*630***	**$67,552,874**

### Cost of CINSW skin cancer campaign

The total cost of implementing the three campaigns between 2006 and 2013 is estimated at $15.63 million (NPV). Table 4 provides annual expenditure and equivalent per capita expenditure. Annual funding ranged from $1.338 million in 2006–07 to $3.678 million in 2010–11, equivalent to per capita expenditure of $0.20 and $0.51, respectively. Expenditure on production and advertising was the key cost driver, accounting for an average 80% of all campaign costs each year.

**Table 4:** Cost of CINSW public education mass media campaigns, 2006–07 to 2012–13 (constant 2010–11 dollars).

	2006–07	2007–08	2008–09	2009–10	2010–11	2011–12	2012–13
Campaign expenditure ($m)	$1.338	$2.583	$2.060	$2.266	$3.678	$2.797	$2.500
Population NSW (m)	6,816	6,885	6,976	7,070	7,145	7,211	7,288
Per capita expenditure	$0.20	$0.38	$0.30	$0.32	$0.51	$0.39	$0.34

### Benefit cost analysis

Comparing the total combined economic NPV of averting skin cancer cases and deaths ($60.17 million) with cost of CINSW campaigns ($15.63 million), results in a benefit cost ratio (BCR) of 3.85 suggesting that for every $1 invested a return of $3.85 is achieved.

### Sensitivity analysis

Varying key assumptions resulted in a BCR range from 2.69 to 5.40 (Table 5). Applying the lower confidence limit of the RR between sunburn and skin cancer results in a BCR of 2.69. Extending the potential benefits of the campaigns to the period 2006–2020 results in a BCR of 5.40. Adjusting potential benefits of the campaigns by a 5 year lagged effect results in a BCR of 4.54.

**Table 5:** Sensitivity analysis.

Analysis	Result
**Base case**	
Cost campaign (2006–2013)—NPV	$15,634,946
Relative risk—1.91 Mel; 1.4 BCC, 1.23 SCC	
Melanoma cases and deaths averted (2006–2013)	885 cases, 109 deaths
NMSC cases and deaths averted (2006–2013)	12,289 cases, 2.6 deaths
Economic value of benefit—NPV	$60,167,541
Benefit cost ratio	3.85
**Sensitivity 1—Lower relative risk**	
Cost campaign (2006–2013)—NPV	$15,634,946
Melanoma cases and deaths averted	687 cases, 85 deaths
NMSC cases and deaths averted (2006–2013)	7,452 cases, 0 deaths
Economic value of benefit—NPV	$42,133,964
Benefit cost ratio	2.69
**Sensitivity 2 –Benefit until 2020**	
Cost campaign (2006–2013)—NPV	$15,634,946
Melanoma cases and deaths averted (2006–2020)	1,307 cases, 161 deaths
NMSC cases and deaths averted (2006–2020)	18,198 cases, 5 deaths
Economic value of benefit—NPV	$84,435,817
Benefit cost ratio	5.40
**Sensitivity 3–5 year lag effect to benefits**	
Cost campaign (2006–2013)—NPV	$15,634,946
Melanoma cases and deaths averted (2011–2020)	1,272 cases, 157 deaths
NMSC cases and deaths averted (2011–2020)	17,949 cases, 3.9 deaths
Economic value of benefit—NPV	$71,010,001
Benefit cost ratio	4.54

Notes: NPV = net present value.

## Discussion

### Limitations

In the absence of better data, we assumed historical trends in melanoma were a reasonable proxy for future cases of melanoma and NMSC. It is acknowledged that there may be differences in melanoma and NMSC trends which would impact on the accuracy of projections. Our inclusion of four age cohorts attempted to provide more accurate projections by considering the variations in historical trends among people of differing age. Counterfactual sun-burn rates were based on an assumption of no campaign exposure, even though CINSW campaigns target the whole NSW population, as do other National and State strategies that may have been operating concurrently. This means that the specific effect of the three CINSW campaigns on rates of sunburn, and therefore rates of melanoma and NMSC, may be over-estimated. Nevertheless, the counterfactual (no intervention) was modelled based on trends prior to the commencement of the three CINSW campaigns which would already have some exposure to national and other state strategies. Therefore, it is reasonable to argue that this analysis estimates the additional impact of the three CINSW campaigns, over and above existing other campaigns. The benefits of the campaigns are also assumed to be immediate. If there was in fact a time lag then potential benefits would not be seen until later in life. Although a sensitivity analysis tested a 5 year lag, analysis of longer lags would require extended modelling of skin cancer incidence past 2020, creating additional uncertainty. Despite the uncertainty from these limitations, the scenario analyses allowed for variation in key assumptions, and the results ranged from a BCR of 2.69 to 5.40.

### Main finding

The three CINSW sun protection campaigns were associated with decreasing proportions of NSW residents reporting lifetime rates of sunburn. Lower sunburn rates translate into fewer skin cancer cases and averted deaths. Over the 8 year period (2006–2013) the results suggest that the cumulative effect of the three campaigns was 885 fewer melanoma cases, 12,289 fewer NMSC cases, 109 melanoma deaths averted and 2.7 NMSC deaths averted. The economic net present value of averting this disease burden is estimated to be $60.17 million. Compared with the $15.63 million net present value cost of delivering the campaigns, there is an estimated BCR of 3.85 suggesting that for every $1 invested a return of $3.85 is achieved.

### Interpretation

The results are broadly consistent with other studies examining the economics of prevention strategies. Although different methodologies were used, Shih et al (2009) reported a BCR of 2.30 [10] for the SunSmart program, Kyle et al (2012) reported a BCR of between 1.95 and 4.02 for a school-based sun safety education program called SunWise [21] and Hirst et al (2012) reported an incremental cost effectiveness ratio of AU$40,890 per quality adjusted life year gained for daily use of sunscreen.

## Conclusion

One of the key objectives of the Skin Cancer Prevention Strategy for NSW (2012–2015) is to increase and utilise evidence to inform future planning and development of skin cancer prevention strategies. This analysis has demonstrated that the CINSW skin cancer public education mass media campaigns provides the community with a positive rate of return on the investment in prevention. The campaigns reduce skin cancer morbidity, mortality and contribute to reducing the economic burden of skin cancer in NSW. The results provide evidence to justify both previous and future campaign expenditure aimed at reducing rates of skin cancer in NSW. The analysis also provides a benchmark against which future campaigns can be judged to establish their relative economic benefits and costs, or estimate the economic efficiency of new forms of social marketing techniques relative to traditional TV and radio advertising.

## Supporting Information

S1 File (Supporting information). NSW cohort specific rates of melanoma (cases per 100,000 persons), 1995–2005 (Table A). Economic and epidemiological parameters used to value impact of campaign on averting skin cancer incidence and mortality (Table B). NSW projected cohort specific rates melanoma (cases per 100,000 persons), 2006–2020 (Table C). NSW projected cohort specific rates BCC (cases per 100,000 persons), 2006–2020 (Table D). NSW projected cohort specific rates SCC (cases per 100,000 persons), 2006–2020 (Table E). Economic value of averting melanoma cases and deaths, 2006–2013 (Table F). Economic value of averting BCC cases, 2006–2013 (Table G). Economic value of averting SCC cases and deaths, 2006–2013 (Table H).
(DOCX)
