# Supplementary material for: Benefit Cost Analysis of Three Skin Cancer Public Education Mass-Media Campaigns Implemented in New South Wales, Australia
Source: PLoS One. 2016 Jan 29;11(1):e0147665. doi: 10.1371/journal.pone.0147665 (PMC4732951; doi:10.1371/journal.pone.0147665)
Supplement: S1 File — NSW cohort specific rates of melanoma (cases per 100,000 persons), 1995–2005 (Table A). Economic and epidemiological parameters used to value impact of campaign on averting skin cancer incidence and mortality (Table B). NSW projected cohort specific rates melanoma (cases per 100,000 persons), 2006–2020 (Table C). NSW projected cohort specific rates BCC (cases per 100,000 persons), 2006–2020 (Table D). NSW projected cohort specific rates SCC (cases per 100,000 persons), 2006–2020 (Table E). Economic value of averting melanoma cases and deaths, 2006–2013 (Table F). Economic value of averting BCC cases, 2006–2013 (Table G). Economic value of averting SCC cases and deaths, 2006–2013 (Table H). (DOCX) [file pone.0147665.s001.docx]

# Supporting information

**Table A: NSW cohort specific rates of melanoma (cases per 100,000 persons), 1995- 2005**

| **Year** | **<25** | **25-49** | **50-75** | **75+** |
| --- | --- | --- | --- | --- |
| 1995 | 3.1 | 29.7 | 90.7 | 145.5 |
| 1996 | 3.7 | 30.2 | 92.8 | 159.7 |
| 1997 | 4.0 | 33.1 | 101.9 | 159.8 |
| 1998 | 3.0 | 31.4 | 94.0 | 154.1 |
| 1999 | 3.1 | 31.9 | 96.1 | 154.2 |
| 2000 | 2.3 | 31.5 | 94.9 | 163.5 |
| 2001 | 2.4 | 30.3 | 101.3 | 174.9 |
| 2002 | 3.4 | 31.8 | 113.0 | 182.4 |
| 2003 | 3.0 | 33.2 | 102.5 | 193.6 |
| 2004 | 3.1 | 33.6 | 109.6 | 195.5 |
| 2005 | 2.3 | 33.2 | 108.4 | 220.2 |

**Table B: Economic and epidemiological parameters used to value impact of campaign on averting skin cancer incidence and mortality**

| **Variable** | **Melanoma** | **NMSC** |
| --- | --- | --- |
| Direct costs per case | $10,230 | $2,336 |
| Indirect morbidity cost per case | $26,082 | $90 |
| Indirect premature mortality (before the age of 65 years) cost per case | $139,076 | $45,355 |
| Average years of life lost per death | 9.58 | 4.62 |
| Average years of life lost per death <65years | 4.83 | 1.41 |

*Source: Doran et al (2015)[*[*19*](#_ENREF_19)*]*

**Table C: NSW projected cohort specific rates melanoma (cases per 100,000 persons), 2006-2020**

| **Year** | **<25** | **25-49** | **50-75** | **75+** |
| --- | --- | --- | --- | --- |
| 2006 | 2.5 | 33.5 | 111.4 | 211.0 |
| 2007 | 2.5 | 33.8 | 113.2 | 217.3 |
| 2008 | 2.4 | 34.0 | 115.1 | 223.6 |
| 2009 | 2.3 | 34.3 | 116.9 | 230.0 |
| 2010 | 2.2 | 34.6 | 118.7 | 236.3 |
| 2011 | 2.1 | 34.9 | 120.5 | 242.6 |
| 2012 | 2.0 | 35.2 | 122.4 | 248.9 |
| 2013 | 1.9 | 35.4 | 124.2 | 255.3 |
| 2014 | 1.9 | 35.7 | 126.0 | 261.6 |
| 2015 | 1.8 | 36.0 | 127.8 | 267.9 |
| 2016 | 1.7 | 36.3 | 129.7 | 274.2 |
| 2017 | 1.6 | 36.6 | 131.5 | 280.6 |
| 2018 | 1.5 | 36.8 | 133.3 | 286.9 |
| 2019 | 1.4 | 37.1 | 135.1 | 293.2 |
| 2020 | 1.4 | 37.4 | 137.0 | 299.5 |

**Table D: NSW projected cohort specific rates BCC (cases per 100,000 persons), 2006-2020**

| **Year** | **<25** | **25-49** | **50-74** | **75+** |
| --- | --- | --- | --- | --- |
| 2006 | 9.4 | 673.6 | 2894.4 | 5241.3 |
| 2007 | 9.1 | 679.3 | 2941.8 | 5398.4 |
| 2008 | 8.8 | 684.9 | 2989.2 | 5555.5 |
| 2009 | 8.5 | 690.5 | 3036.7 | 5712.6 |
| 2010 | 8.1 | 696.1 | 3084.1 | 5869.8 |
| 2011 | 7.8 | 701.7 | 3131.6 | 6026.9 |
| 2012 | 7.5 | 707.3 | 3179.0 | 6184.0 |
| 2013 | 7.2 | 713.0 | 3226.5 | 6341.1 |
| 2014 | 6.9 | 718.6 | 3273.9 | 6498.3 |
| 2015 | 6.6 | 724.2 | 3321.4 | 6655.4 |
| 2016 | 6.3 | 729.8 | 3368.8 | 6812.5 |
| 2017 | 6.0 | 735.4 | 3416.3 | 6969.6 |
| 2018 | 5.7 | 741.0 | 3463.7 | 7126.8 |
| 2019 | 5.3 | 746.7 | 3511.2 | 7283.9 |
| 2020 | 5.0 | 752.3 | 3558.6 | 7441.0 |

**Table E: NSW projected cohort specific rates SCC (cases per 100,000 persons), 2006-2020**

| **Year** | **25-49** | **50-74** | **75+** |
| --- | --- | --- | --- |
| 2006 | 180.7 | 1,359.2 | 2,932.9 |
| 2007 | 182.2 | 1,381.5 | 3,020.8 |
| 2008 | 183.7 | 1,403.8 | 3,108.7 |
| 2009 | 185.2 | 1,426.1 | 3,196.7 |
| 2010 | 186.7 | 1,448.4 | 3,284.6 |
| 2011 | 188.2 | 1,470.6 | 3,372.5 |
| 2012 | 189.7 | 1,492.9 | 3,460.4 |
| 2013 | 191.2 | 1,515.2 | 3,548.3 |
| 2014 | 192.7 | 1,537.5 | 3,636.3 |
| 2015 | 194.2 | 1,559.8 | 3,724.2 |
| 2016 | 195.7 | 1,582.1 | 3,812.1 |
| 2017 | 197.2 | 1,604.3 | 3,900.0 |
| 2018 | 198.7 | 1,626.6 | 3,988.0 |
| 2019 | 200.3 | 1,648.9 | 4,075.9 |
| 2020 | 201.8 | 1,671.2 | 4,163.8 |

*NB: There were no cases for the <25 age group.*

**Table F: Economic value of averting melanoma cases and deaths, 2006-2013**

| **Year** | **Direct cost savings** | **Indirect cost savings - morbidity** | **Indirect cost savings - premature mortality** | **Total indirect costs savings** | **Total** |
| --- | --- | --- | --- | --- | --- |
| 2006 | $847,630 | $2,161,071 | $519,123 | $2,680,193 | $3,527,823 |
| 2007 | $949,827 | $2,421,626 | $581,712 | $3,003,338 | $3,953,165 |
| 2008 | $1,036,460 | $2,642,501 | $634,770 | $3,277,271 | $4,313,731 |
| 2009 | $1,113,188 | $2,838,123 | $681,761 | $3,519,883 | $4,633,071 |
| 2010 | $1,183,196 | $3,016,612 | $724,637 | $3,741,249 | $4,924,445 |
| 2011 | $1,247,972 | $3,181,762 | $764,308 | $3,946,070 | $5,194,043 |
| 2012 | $1,308,929 | $3,337,174 | $801,641 | $4,138,814 | $5,447,743 |
| 2013 | $1,369,431 | $3,491,425 | $838,694 | $4,330,119 | $5,699,550 |
| ***Total*** | **$9,056,633** | **$23,090,293** | **$5,546,646** | **$28,636,939** | **$37,693,573** |

**Table G: Economic value of averting BCC cases, 2006-2013**

| **Year** | **Direct cost savings** | **Indirect cost savings - morbidity** | **Indirect cost savings - premature mortality** | **Total indirect cost savings** | **Total** |
| --- | --- | --- | --- | --- | --- |
| 2006 | $2,209,394 | $85,398 | $0 | $85,398 | $2,294,792 |
| 2007 | $2,479,682 | $95,845 | $0 | $95,845 | $2,575,528 |
| 2008 | $2,708,834 | $104,703 | $0 | $104,703 | $2,813,537 |
| 2009 | $2,912,385 | $112,570 | $0 | $112,570 | $3,024,955 |
| 2010 | $3,100,023 | $119,823 | $0 | $119,823 | $3,219,846 |
| 2011 | $3,275,245 | $126,596 | $0 | $126,596 | $3,401,841 |
| 2012 | $3,440,369 | $132,978 | $0 | $132,978 | $3,573,347 |
| 2013 | $3,604,544 | $139,324 | $0 | $139,324 | $3,743,868 |
| ***Total*** | **$23,730,476** | **$917,237** | **$0** | **$917,237** | **$24,647,713** |

**Table H: Economic value of averting SCC cases and deaths, 2006-2013**

| **Year** | **Direct cost savings** | **Indirect cost savings – morbidity** | **Indirect cost savings – premature mortality** | **Total indirect cost savings** | **Total** |
| --- | --- | --- | --- | --- | --- |
| 2006 | $461,506 | $17,838 | $4,096 | $21,934 | $483,441 |
| 2007 | $518,244 | $20,031 | $4,599 | $24,631 | $542,875 |
| 2008 | $566,527 | $21,898 | $5,028 | $26,925 | $593,452 |
| 2009 | $609,594 | $23,562 | $5,410 | $28,972 | $638,567 |
| 2010 | $649,768 | $25,115 | $5,767 | $30,882 | $680,649 |
| 2011 | $687,670 | $26,580 | $6,103 | $32,683 | $720,353 |
| 2012 | $723,251 | $27,955 | $6,419 | $34,374 | $757,625 |
| 2013 | $758,574 | $29,321 | $6,732 | $36,053 | $794,626 |
| ***Total*** | **$4,975,135** | **$192,300** | **$44,153** | **$236,453** | **$5,211,588** |
